# Near-Infrared Spectroscopy and Machine Learning for Fast Quality Prediction of Bottle Gourd

**DOI:** 10.3390/foods14142503

**Published:** 2025-07-17

**Authors:** Xiao Guo, Hongyu Huang, Haiyan Wang, Chang Cai, Ying Wang, Xiaohua Wu, Jian Wang, Baogen Wang, Biao Zhu, Yun Xiang

**Affiliations:** 1 College of Horticulture Science, Zhejiang A&F University, Hangzhou 311300, China; m15034454280@163.com (X.G.); billzhu@zafu.edu.cn (B.Z.); 2 Institute of Vegetables, Zhejiang Academy of Agricultural Sciences, Hangzhou 310021, China; why20200304@163.com (H.W.); wangying@zaas.ac.cn (Y.W.); wuxiaohua2001@126.com (X.W.); wangjian@zaas.as.cn (J.W.); centuraldragon@sina.com (B.W.); 3 Institute of Cyberspace Security, Zhejiang University of Technology, Hangzhou 310023, China; hhycrispin@163.com (H.H.); caichang199891@gmail.com (C.C.); 4 Key Laboratory of Vegetable Germplasm Innovation and Quality Breeding in the Province, Hangzhou 310021, China; 5 State Key Laboratory for Quality and Safety of Agro-Products, Hangzhou 310021, China

**Keywords:** bottle gourd, protein and amino acid content, near-infrared spectroscopy, machine learning, quality prediction

## Abstract

Protein and amino acid content are the crucial quality parameters in bottle gourd, and traditional measurement methods for detecting those parameters are complicated, time-consuming, and costly. In this study, we employed NIRS along with machine learning and neural network-based methods to model and predict protein and free amino acids (FAAs) of bottle gourd. Specifically, the content of protein and FAAs were measured through conventional methods. Then a near-infrared analyzer was utilized to obtain the spectral data, which were processed using multiple scattering correction (MSC) and standard normalized variate (SNV). The processed spectral data were further processed using feature importance selection to select the feature bands that had the highest correlation with protein and FAAs, respectively. The models for protein and FAAs estimation were developed using support vector regression (SVR), ridge regression (RR), random forest regression (RFR), and fully connected neural networks (FCNNs). Among them, ridge regression achieved the optimal performance, with determination coefficients (R^2^) of 0.96 and 0.77 on the protein and FAAs test sets, respectively, and root mean square error (RMSE) values of 0.23 and 0.5, respectively. Based on this, we developed a precise and rapid prediction model for the important quality indices of bottle gourd.

## 1. Introduction

Bottle gourd (*Lagenaria siceraria* (Mol.) Standl.), also known as white flowered gourd, is a cultivated variety that belongs to the Cucurbitaceae family. It is an annual climbing herbaceous plant that usually blooms in the evening [1]. As a vegetable, its fruit is the economic organ of the bottle gourd, which is rich in protein, a wide range of amino acids, vitamins, and trace elements. The flesh of bottle gourd is characterized by its fine texture, white color, tenderness, delightful aroma, and delicious flavor, making it highly popular among consumers [2].

Protein and free amino acids (FAAs) represent crucial quality indicators for bottle gourd [3]. However, the conventional detection methods used for assessing bottle gourd protein and amino acids have limitations, such as lengthy detection times, low efficiency, and high costs, making them unsuitable for mass detection in production practices.

In recent years, hyperspectral imaging (HSI), NIRS, ultrasound testing, and electrical characteristic testing have been widely used for the rapid detection of fruit and vegetable quality [4]. Due to its convenience, environmental friendliness, and stability, NIRS technology has gradually become the mainstream approach for the rapid detection of agricultural products [5].

The NIRS wavelength range (780~2526 cm^−1^) is between visible and midinfrared light spectra. By recording the harmonic and combination frequency absorption of hydrogen-containing groups (C-H, N-H, and O-H), NIRS can present different absorption peaks at different wavelengths [6]. When using NIRS technology to perform quality analysis of fruits and vegetables, a highly stable and precise mathematical model should be constructed by mining spectral information data and chemometrics methods [7,8,9,10].

For example, Chen et al. [11] utilized CARS, Random Frog, and the Continuous Projection Algorithm to carry out dimensionality reduction on NIRS spectral data to quantitatively predicting the content of Rg1 and Rc in ginsenosides. The determination coefficients of the two models were both above 0.94. In predicting the content of linear chain starch in sweet potatoes, Masithoh et al. [12] collected NIRS spectral information from two bands and employed the partial least squares (PLS) to construct a prediction model for the linear chain starch content, linear chain starch percentage, total starch content, and thermal characteristics. The results showed that the R^2^ values of various starch indicators ranged from 0.85 to 0.92. When predicting the soluble solids content (SSC) of strawberries, AgulheiroSantos et al. [13] used PLS to perform a quantitative analysis of the strawberry SSC after the first-order derivative of the spectrum. The determination coefficients of the calibration set and prediction set were 0.9277 and 0.8207, respectively. Moreover, Wold et al. [14] used NIR to detect the soluble solids and lycopene content of cherry tomato with the diffuse reflecting spectrum. Somton et al. [15] developed a partial least squares regression (PLSR) model to classify durian maturity based on peel and stem absorbance. Zhou et al. [16] collected NIR spectral data of figs and clustered them into various quality indicators. However, there are no studies related to establishing a rapid prediction method for bottle gourd protein and FAAs. Consequently, there is an urgent need to develop a rapid detection technology specifically for bottle gourd protein and amino acids to enable quick and efficient detection.

In this study, 206 samples of bottle gourd fruit were divided into two portions. One part was measured for the protein and FAA content using traditional measurement methods, while the other part was processed through spectral data acquisition with a near-infrared analyzer. The support vector regression, ridge regression, random forest regression, deep neural network, and other algorithms were used to compare and construct rapid prediction models for the protein and FAA content in bottle gourd. The results demonstrated that the spectral data preprocessed by CARS could accurately estimate protein and FAA concentrations by constructing appropriate models, with determination coefficient R^2^ values of 0.96 and 0.77, and RMSE values of 0.23 and 0.5, respectively. Based on this, a rapid assessment technology for important quality indicators, such as the content protein and amino acids, in bottle gourd was developed.

## 2. Materials and Methods

### 2.1. Sample Preparation

**Figure 1:**
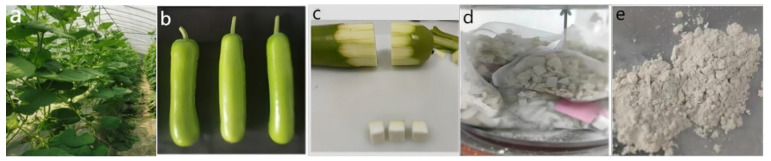
(**a**) Bottle gourd experimental materials planted in greenhouse; (**b**) bottle gourd fruits at commercial maturity stage; (**c**) the middle section of the fruit flesh of bottle gourd; (**d**) the frozen sample of bottle gourd at low temperature; (**e**) the powdered bottle gourd obtained by grinding.

The bottle gourd crop was cultivated at the Yangdu Scientific Research and Innovation Base of the Zhejiang Academy of Agricultural Sciences (30°30′76.63″ N, 120°19′55.16″ E). They were grown in the greenhouse (28 °C/20 °C day/night, 80% relative humidity, and a 16 h light/8 h dark photoperiod) from March to August 2023, with a spacing of 80 cm between the rows and 50 cm between the plants. Figure 1a shows the planting layout inside the facility.

A total of 206 bottle gourd core germplasm populations were selected as test materials, all of which were inbred lines [17]. During the period of commercial maturity (60 d from sowing) of the bottle gourd, uniform and tender fruits were harvested (Figure 1b). Each sample consisted of 9 commercial fruits, with 3 fruits in each group. The middle section of the fruit was sampled from each group (Figure 1c). The samples were freeze-dried in a freeze-dryer (LGJ-10) for 3–4 days (Figure 1d) and then grounded into powder using a high-speed grinder (JXFSTPRP-24L) (Figure 1e).

Each numbered sample was divided into the following three parts.

#### 2.1.1. Protein Determination

The first part was mixed with sulfuric acid and the protein content of each sample was determined using a K1100 fully automatic Kjeldahl nitrogen analyzer.

#### 2.1.2. Free Amino Acids Determination

The second part was analyzed for FAA content using a Hitachi L-8900 Amino Acid Analyzer (Hitachi Co., Tokyo, Japan).

#### 2.1.3. Near-Infrared Spectral Scanning

The remaining part was scanned using an ANTARIS II Fourier Transform Near Infrared (FT-NIR) Analyzer to obtain near-infrared spectral data.

In this study, the entire collection of samples underwent both near-infrared and traditional analyses. Moreover, the datasets were randomly divided into a training set and a testing set with an 8:2 ratio. In general, 164 training samples and 42 test samples were utilized.

### 2.2. Vis–NIR Spectra Acquisition and Preprocessing

**Figure 2:**
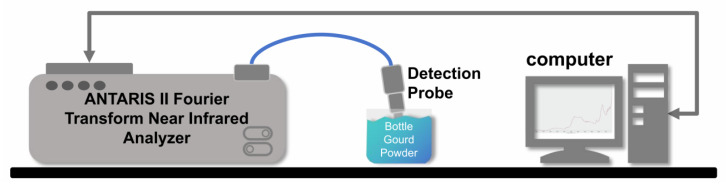
ANTARIS II Fourier Transform Near Infrared Analyzer.

To acquire the NIR spectra of bottle gourd, the Thermo Nicolet ANTARIS II Fourier Transform Near Infrared (FT-NIR) Analyzer, as shown in Figure 2, was used. The scanning was performed at a constant temperature of 24 °C and a humidity of 60%. The scanning spectral range was from 1000 to 2500 cm^−1^, with a total of 64 scans per sample and a resolution of 8 cm^−1^. Before scanning, the sample was loaded into a sample cup with a diameter of 5 cm and a sample height of approximately 1.5 cm. The samples were maintained at a consistent thickness and compaction to minimize measurement errors caused by non-uniform sample loading. Each sample was scanned three times, and the average absorption spectrum was calculated as the spectral value. The near-infrared spectral information of all samples was collected and stored.

To eliminate the background, noise, and interference caused by instrument and placement variations during data acquisition, it was necessary to preprocess the raw spectra. Common preprocessing techniques included data smoothing, derivatives, and scatter correction. The primary purposes of smoothing and derivatives were to reduce the impact of noise and enhance the signal-to-noise ratio. Scatter correction was employed to eliminate the scattering effects caused by particles of different sizes.

By applying the multiple scattering correction (MSC) and standard normalized variate (SNV) methods to preprocess the raw spectral data, the interference from noise and baseline drift could be eliminated. The MSC method corrected the effects of multiple scattering, which led to an increase in the optical path length and a decrease in the signal intensity, by constructing the ratio of the sample spectrum to a reference spectrum. The basic formula for MSC correction is as follows:

(1) $$MSC=\frac{R_{sample}-R_{min}}{R_{ref}-R_{min}}$$

where *R_sample_* is the sample spectrum, *R_ref_* is the reference spectrum, and *R_min_* is the minimum value of the spectrum. The purpose of *MSC* correction is to remove the residual scattering component of the spectrum and improve the accuracy and comparability of the sample spectrum.

Variable Standardization (*SNV*) is a normalization technique to eliminate variations caused by differences in light intensity and baseline drift. The *SNV* formula is as follows:

(2) $$SNV=\frac{X-\mu }{\sigma }$$

where *X* is the original spectral data, *µ* is the mean, and Σ is the standard deviation of the spectral data. For each spectrum, the *SNV* method subtracts the mean from each data point in the spectrum and divides it by the standard deviation. This process results in a processed spectrum with mean value of 0 and a standard deviation of 1. In that case, the overall intensity changes and baseline drift in the spectrum are eliminated, and thus the spectral features are highlighted.

### 2.3. Feature Bands Selection

Competitive Adaptive Reweighted Sampling (CARS), which was based on Monte Carlo sampling and PLS regression coefficients, was widely used for feature band optimization of near-infrared spectral data [18]. CARS was employed to sample the near-infrared spectral data after preprocessing. In each iteration, the calibration set samples were randomly re-selected with an exponential decay function to facilitate the selection of bands. The data were optimized using the adaptive reweighted method. The subset with the minimum cross-validation root mean square error was selected as the feature band. The sklearn library in Python 3.10 was used to implement the algorithm and extract the feature bands from the preprocessed near-infrared spectral data.

### 2.4. Regression Model Based on Ridge Regression

In order to predict the protein and FAAs of the bottle gourd sample through spectral information, several common regression models were selected for experimentation. They were used to establish the relationship between input (spectral information) and output (protein and FAA content), thereby enabling the prediction of unknown samples. Those common methods included ridge regression [19], random forest regression [20,21], support vector machine regression [22,23], ordinary least squares, and neural networks. Ridge regression was an extension of linear regression that addresses the issue of multicollinearity by imposing constraints on the regression coefficients. Ridge regression worked better for relatively smaller datasets. When the dataset was small, random forest, neural networks, and support vector machines could be overly flexible, prone to overfitting, and less responsive to variable correlations. Therefore, considering the high correlation between the feature bands and the limited data samples, the ridge regression-based approach was adopted for constructing the prediction model.

The objective of ridge regression was to solve for a coefficient vector *β* of size *p ×* 1 that minimizes the following loss function.

Algorithm Feature Selection Algorithm:

Dataset Splitting:
Split the calibration dataset into a modeling set (80%) and a prediction set (20%).
Monte Carlo Sampling Iterations N setting:
Define the number of Monte Carlo sampling iterations as N.

Iterative Process:

For each iteration i = 1 to N, the following steps were carried out:

1. Randomly select a portion of the modeling set for modeling, with the remaining samples reserved for validation.
2. Build a PLS model on the selected portion of the modeling set.
3. Calculate the absolute values of the regression coefficients for each variable in the PLS model $\left|b_{i}\right|$.
4. Compute the weight for each variable $\left|\omega_{i}\right|$ using the formula $\omega_{i}=\frac{\left|b_{i}\right|}{\sum \left|b_{i}\right|}$, where m is the number of remaining variables.
5. Calculate the retention ratio for the wavelength points using an exponential decay function (EDF), as follows:
  (3) $$\mu ={\left(\frac{n}{2}\right)}^{\frac{1}{N-1}},\;k=\frac{\ln\left(\frac{n}{2}\right)}{N-1}$$
  (4) $$R_{i}=\left\{\begin{array}{c} 1,if\;i=1\\ \mu \cdot \exp\left(-k\cdot i\right),if\;i\neq 1\;and\;i\neq N\\ \frac{2}{n},\;if\;i=N \end{array}\right.$$
  where *n* is the number of variables.
6. Use AWS to select $R_{i}\cdot n$ wavelength variables for the next modeling round.
7. Construct a PLS model on the selected features and calculate the root mean square error of cross-validation (RMSECV).

Final Feature Subset Selection:

From the N sampling iterations, select the subset of wavelength features with the smallest RMSECV value as the final feature subset with the following loss function:

(5) $$L\left(\beta \right)={\left|y-X\beta \right|}^{2}+\alpha {\left|\beta \right|}^{2}$$

where *| · |* denotes the L2 norm, *y − Xβ* is the residual vector, and *α|β|^2^* is the regularization term, with *α* as the regularization parameter controlling its strength. By minimizing the loss function, we can obtain the estimated coefficient vector *β*. The closed-form solution for *β* is as follows:

(6) $${\hat{\beta }}_{ridge}={\left(X^{T}X+\alpha I\right)}^{-1}X^{T}y$$

where $X^{T}$ represents the transpose of X and I is a *p × p* identity matrix.

### 2.5. Evaluation Criteria

The entire dataset consists of 206 near-infrared spectroscopy data points, which were randomly divided into the following two parts: a training set (80%, 164 data points) and testing set (20%, 42 data points). At the same time, we constructed ridge regression, random forest regression, support vector machine regression, and a fully connected neural network for comparison.

The coefficient of determination *R*^2^ and *RMSE* were used as evaluation metrics, which were defined by Equations (7) and (8), respectively. *R*^2^ represented the goodness of the model fit and ranged from 0.0 to 1.0, where 1.0 indicated a perfect linear relationship between the observed and predicted values. A lower *RMSE* indicated a better fit of the model to the dataset.

(7) $$R^{2}=1-\frac{\sum_{i}{\left({\hat{y}}_{i}-y_{i}\right)}^{2}}{\sum_{i}{\left(\bar{y}-y_{i}\right)}^{2}}$$

(8) $$RMSE=\sqrt{\frac{1}{m}\sum_{1}^{m}{\left(y_{i}-{\hat{y}}_{i}\right)}^{2}}$$

## 3. Results

### 3.1. NIR Spectral Data and Preprocessing Results

**Figure 3:**
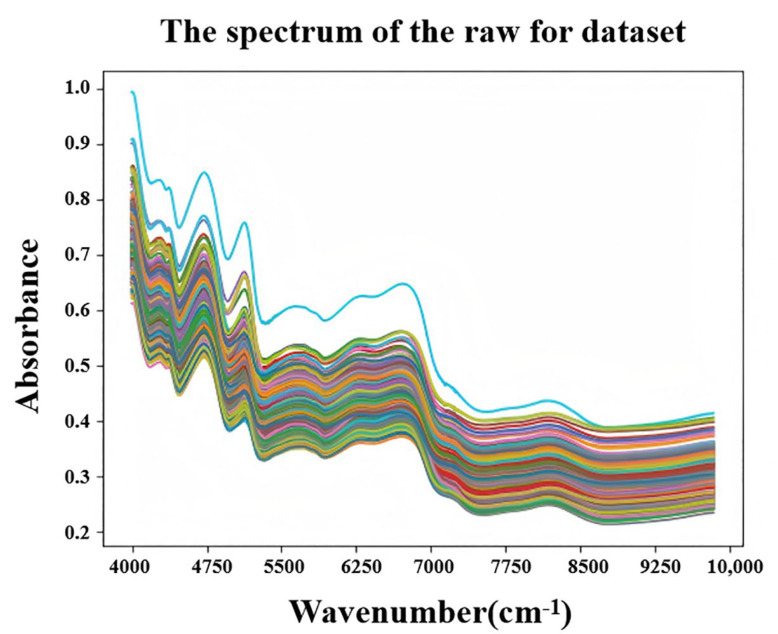
Raw unprocessed NIR data.

As shown in Figure 3, the near-infrared (NIR) reflectance spectra of 206 core germplasm populations of bottle gourd within the spectrum ranged from 4000 to 10,000 cm^−1^, and the spectral trends of all of the samples were similar. In terms of reflectance, there were significant absorption peaks at 4698 cm^−1^, 5102 cm^−1^, and 6697 cm^−1^. Existing studies [24,25,26] demonstrated that NIR absorption peaks at wavelengths of 10416 cm^−1^ and 7042 cm^−1^ were closely related to the water content; the absorption peaks at 6802 cm^−1^, 6756 cm^−1^, and 6667 cm^−1^ were related to the protein content; and the absorption at 8333 cm^−1^, 8130 cm^−1^, 7633 cm^−1^, 7352 cm^−1^, 6211 cm^−1^, and 5882 cm^−1^ was related to the carbohydrate content.

**Figure 4:**
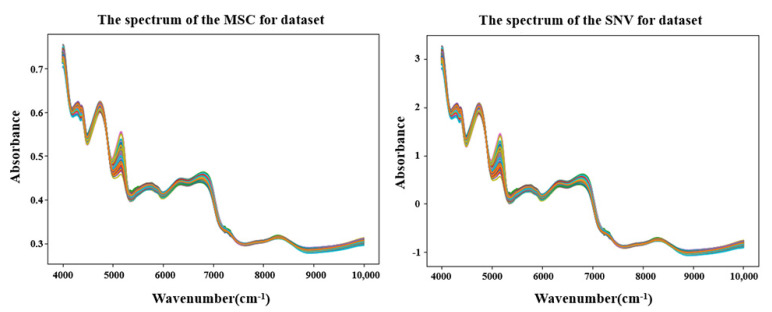
Preprocessing operations on NIR data using SNV and MSC.

According to the indications in Figure 3 and Figure 4, distinct peaks in the reflectance spectrum appeared around wavenumbers of 6700 cm^−1^, 5100 cm^−1^, and 4700 cm^−1^. Therefore, by combining this information with the preceding content, we inferred that the protein absorption peak was around 6700 cm^−1^, and the amino acid absorption peaks were intensively around 4700 cm^−1^ and 5100 cm^−1^.

To reduce the noise and smooth the curves, the original NIR spectra data were preprocessed using the MSC and SNV methods. By comparing the original NIR data, both the MSC and SNV methods were effective in reducing the noise, but there was little difference in terms of the trend between them. The specific results are shown in Figure 4, where SNV showed an overall improvement in absorbance values compared to MSC. Therefore, for the convenience of subsequent experiments, the data processed with MSC were used as the experimental result. However, the preprocessing methods for the NIR spectroscopy data were not limited to MSC and SNV. There are also other commonly used methods, such as Z-transformation, first derivative, and second derivative. Therefore, combining multiple preprocessing methods could be employed to achieve more accurate results.

### 3.2. Feature Band Extraction Results

**Figure 5:**
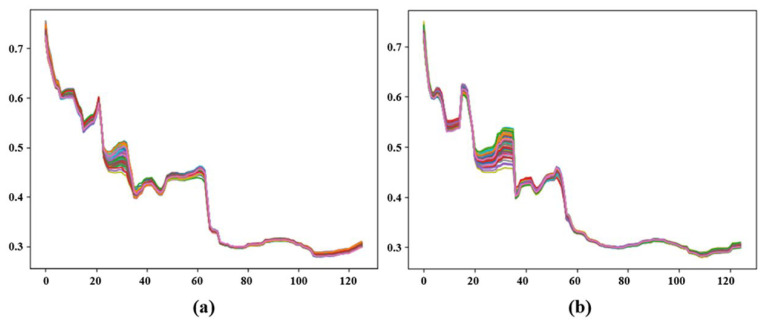
Feature band extraction of preprocessed near-infrared data using Competitive Adaptive Reweighted Sampling algorithm. (**a**) Protein. (**b**) FAAs.

In this work, CARS was employed to extract significant feature bands. Figure 5 illustrates the distribution of these feature bands. From Figure 5a,b, it could be observed that the extracted feature bands were predominantly concentrated between 4000 and 5000 cm^−1^, and 7000 and 10,000 cm^−1^. While there was some discrepancy from the previously mentioned spectral wavenumbers for the protein and FAAs reflectance–absorbance spectra, they generally aligned around these two ranges. Compared to the original near-infrared spectroscopy data, the number of bands were significantly reduced. Through the application of the CARS algorithm, we identified 127 highly correlated bands for protein and 124 for FAAs.

In addition, from the distribution of feature bands represented by the red line in the Figure 5, the feature bands obtained based on CARS were relatively evenly distributed. The adaptive competitive feature extraction algorithm selected and extracted the most representative features through a competitive process, without explicitly favoring specific bands.

### 3.3. Predictive Model Evaluation

**Figure 6:**
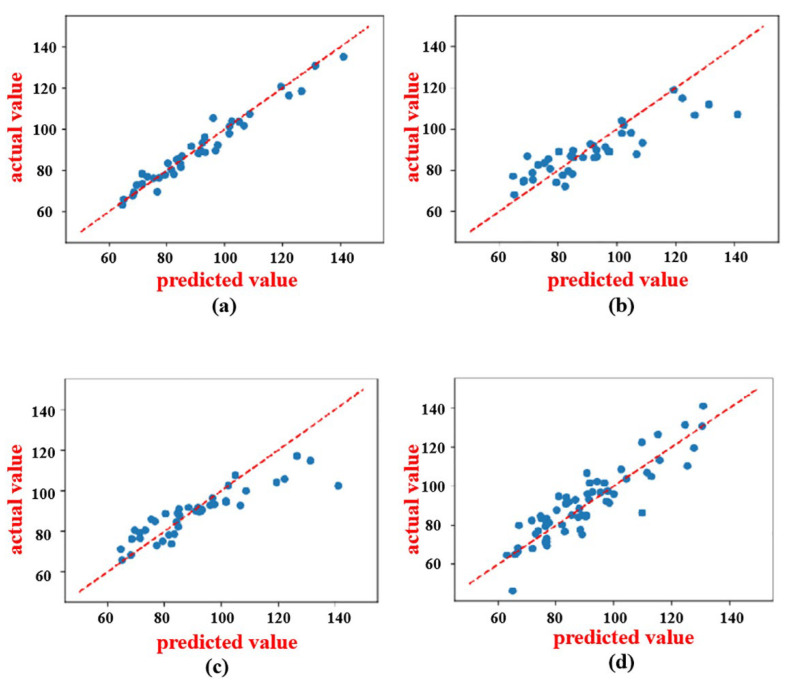
Various prediction models under CARS for protein. (**a**) Ridge regression. (**b**) Random forest. (**c**) Support vector machine. (**d**) FCNN.

**Figure 7:**
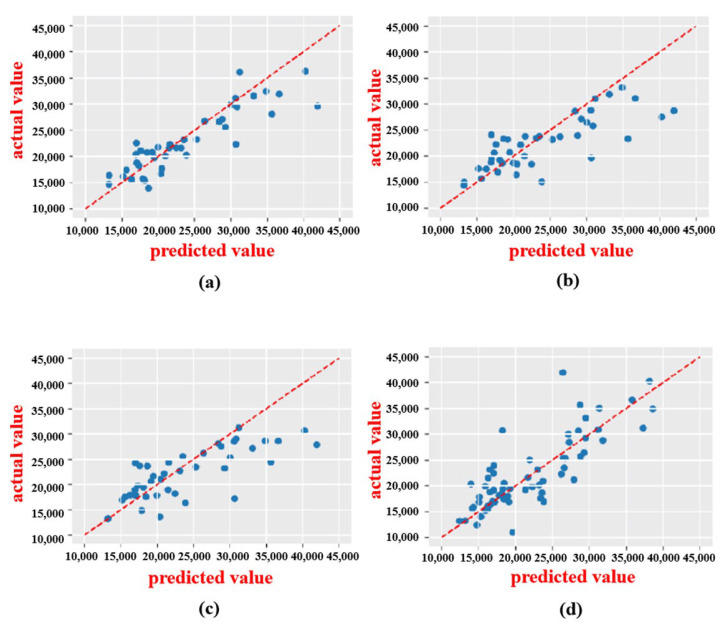
Various prediction models under CARS for FAAs. (**a**) Ridge regression. (**b**) Random forest. (**c**) Support vector machine. (**d**) FCNN.

In this experiment, the support vector machines, random forests, ridge regression, and FCNN were used as the modeling approaches for the protein and FAAs. As shown in Figure 6 and Figure 7, and Table 1 and Table 2, the prediction models for the protein and FAA content based on the ridge regression algorithm exhibited a robust fit to the observed data (test set), with the predicted values closely aligning with true values along the diagonal. Furthermore, in comparison to random forests, support vector machines, and fully connected neural networks, ridge regression demonstrated faster speed and higher *R*^2^ values. The model based on ridge regression had an *R*^2^ value of 0.96 and an *RMSE* value of 0.23 in predicting the protein content, which was 16% superior to the second best. Additionally, in the prediction of the FAA content, it had an *R*^2^ value of 0.77 and an *RMSE* value of 0.5, which was 17% better than the second best.

## 4. Discussion

Upon scrutiny, the observed pattern can be attributed to the correlation between the model performance and the size of dataset. Given that our current dataset consists of just over 200 entries, ridge regression seems to perform exceptionally well in small-sample datasets. The regularization term in ridge regression proves advantageous in stabilizing estimates and preventing overfitting to the training data in such scenarios with limited-samples. Conversely, models such as random forests and neural networks are more appropriate for extensive datasets and intricate relationships. Furthermore, in existing research on the prediction of fruit and vegetable proteins, taking winter wheat and soybeans as examples, Zhu et al. [27] conducted NIR push-broom hyperspectral measurements on both using PLSR for the construction of the calibration model, and finally obtained accurate prediction models for evaluating the proteins in winter wheat and soybeans. For the prediction of winter wheat protein, the calibration correlation coefficient was R = 0.973 (*R*^2^ = 0.947), and the standard error of prediction (SEP) was 0.556. For soybean protein, *R* = 0.902 (*R*^2^ = 0.814) and SEP = 1.332. Additionally, Wang et al. [28] also applied PLSR calibration models to fit the protein content and spectral data of peanuts, and achieved a prediction coefficient of determination of 0.885 and a root mean square error of prediction (RMSEP) of 0.465%. In terms of accuracy, when the sample size was small, the ridge regression algorithm could be used as a rapid method for protein prediction model construction compared to the former approach.

However, there is still room for improvement when compared to the existing predictions of the amino acid content. For example, Guo et al. [29] evaluated matcha quality using near-infrared (NIR) spectroscopy. They utilized SiPLS (Sequential Importance PLS), SPA (Successive Projections Algorithm), GA (Genetic Algorithm), and SA (Simulated Annealing) to construct prediction models for total polyphenols (TPs) and FAAs. The results demonstrated that the SiPLS-SPA and SiPLS-SA models in combination with NIR exhibited higher predictive capabilities, with both Rc and RP values exceeding 0.97.

In the present study, we attempted to detect the internal physicochemical indicators of bottle gourd using HSI and magnetic resonance imaging devices. However, due to factors such as the thickness of the bottle gourd’s skin and volume (which is unable to penetrate thick or dense materials), it was not possible to effectively determine its physicochemical indicators without damage. Therefore, we planned to conduct destructive experiments on a batch of bottle gourd and, in conjunction with machine learning, fit some of the data to predict the internal physicochemical indicators of the remaining samples.

In comparison to traditional chemical testing, the studied method has the advantages of being fast, accurate, and scalable. Firstly, for a batch of bottle gourd, within controllable costs, we can randomly select some samples as an experimental dataset. After necessary processing, we performed near-infrared scans on their powdered particles and tested the required physicochemical indicators through a certified inspection agency. Subsequently, a predictive model was constructed by combining the near-infrared spectral data and physicochemical indicators of the inspected samples. When the test set of the inspected samples exhibited a high degree of fit in the predictive model (i.e., an R^2^ value exceeding 0.9), the model’s predictions could be extended to another bottle gourd in that batch.

However, this method possessed certain limitations. Tissues might suffer loss when fruits were subjected to destructive testing, and there might be inaccuracies in determining certain indicators after drying. Additionally, the presence of gaps and impurities in powdered particles might affect the sensitivity of near-infrared spectroscopy, leading to inaccurate detection results.

The method explored in this study had been successfully applied to the measurement of physicochemical indicators in bottle gourd. However, its significance extends beyond this application; in the future, NIR technology will be applied in more and more fields, such as seed germination [30], and stress-resistant, high-quality bottle gourd variety screening [31]. Such technologies can be generalized to other members of the Cucurbitaceae family as well as soft-skinned fruits. Furthermore, it can be applied during the growth process of bottle gourd to uncover other meaningful features, facilitating the cultivation of new varieties and improving the yield and quality.

## 5. Conclusions

In this work, the estimation modes for physicochemical indicators of the bottle gourd, namely, protein and FAAs, were established based on near-infrared spectroscopy technology and machine learning techniques. Moreover, feature selection using the competitive adaptive reweighted algorithm was utilized to extract the most correlated feature wavelengths. Subsequently, several widely used machine learning models were implemented and compared.

The results demonstrated that the feature wavelengths obtained through the competitive adaptive reweighted algorithm, along with the ridge regression model, achieved an R^2^ of 0.96 and 0.77 for the protein and FAAs, respectively. Therefore, it was concluded that the ridge regression model in combination with near-infrared spectroscopy technologies could effectively estimate the protein and FAA content in bottle gourd.

## Figures and Tables

**Table 1:** Results of the protein prediction model.

	Ridge Regression	Random Forest	Support Vector Machine	FCNN
*R*^2^ (CARS)	0.96	0.78	0.77	0.80
*RMSE* (CARS)	0.23	0.5	0.5	0.5

**Table 2:** Results of the FAAs prediction model.

	Ridge Regression	Random Forest	Support Vector Machine	FCNN
*R*^2^ (CARS)	0.77	0.56	0.52	0.6
*RMSE* (CARS)	0.5	0.7	0.7	0.6

## Data Availability

The original contributions presented in the study are included in the article; further inquiries can be directed to the corresponding author.
